# Long-Term Effects of Sustained Regular Medication in Hypertensive Patients in Yunnan, China: A Cohort Study of 5 Years' Follow-Up

**DOI:** 10.1155/ijhy/4505824

**Published:** 2025-05-08

**Authors:** Min Ma, Huadan Wang, Linhong Pang, Zihong Guo, Manli Sun, Yajing Zhao, Yi Shi, Xia Wu, Junjie Song, Qiuyan Zhu, Lin Duo, Zhongjie Wang, Yu Xia, Liping He, Mingjing Tang

**Affiliations:** ^1^Fuwai Yunnan Cardiovascular Hospital, Affiliated Cardiovascular Hospital of Kunming Medical University, Kunming 650221, China; ^2^Qujing Medical College, Qujing 655011, China; ^3^School of Public Health, Kunming Medical University, Kunming 650500, China; ^4^Yunnan Center for Disease Control and Prevention, Kunming 650100, China

**Keywords:** antihypertensive medication, blood pressure control, hypertension, hypertension management, medication adherence

## Abstract

**Background:** The relationship between different grades of compliance to antihypertensive medication and blood pressure (BP) control rate remains unclear. This study highlighted the Chinese Basic Public Health Service (BPHS) program upgraded antihypertension medication compliance to improve the blood pressure control rate contributing to the UN sustainable development goal of lowering chronic illness fatalities by one-third by 2030.

**Methods:** In 2015, 1254 hypertensive patients aged ≥ 35 years were selected in the baseline survey in Yunnan Province by a multistage stratified random sampling method and followed up from 2018 to 2022. Then, they were divided into three groups by tertiles as antihypertensive medicine compliance “poor,” “intermittent,” and “sustained” groups. Then, the robust variance Poisson regression models were performed to estimate the association between three groups and the number of referrals. Kaplan–Meier curves calculated the cumulative risk of onset and survival probability of cardiovascular events from three medication compliance groups.

**Results:** A total of 1254 hypertension patients were included in the study; 992, 1218, 1121, 1066, and 999 hypertension patients were followed up annually. From the baseline to last follow-up, systolic BP declined with the largest decrease in the sustained group (2015: 152.88 ± 21.64 mmHg vs. 2022: 134.61 ± 10.49 mmHg, *p* < 0.05) and the least decrease in the poor group (2015: 157.07 ± 15.37 mmHg vs. 2022: 140.33 ± 12.04 mmHg, *p* < 0.05). Under the management of BPHS, the BP control rate of all three groups increased significantly. Compared with the baseline, the control rate in the sustained group reached 70% in 2022 (*p* < 0.01). The number of referrals from the poor group was 11.5%, higher compared with the sustained group (IRR = 1.115 and 95% CI: 1.043–1.193). The poor group also had the highest probability of cardiovascular disease (CVD). The survival probability in the sustained group was the highest and kept at 0.950 at the end of 5 years.

**Conclusions:** High-grade compliance to antihypertensive drugs significantly improves BP control rate and reduces the risk of CVD events and mortality. The decline of medication compliance grades is closely related to decreasing BP control rate and increased risk of CVD and mortality.

## 1. Introduction

Hypertension is the leading cause of cardiovascular disease (CVD) and premature death worldwide [1]. In 2019, an estimated 1.28 billion adults aged from 30 to 70 years, worldwide had hypertension, and the hypertension control rate was only 20% [2]. In 2019, hypertension accounted for 19.2% of deaths (10.8 million people) [3]. A total of 245 million Chinese adults aged ≥ 18 years had hypertension, 40.7% of hypertension patients were taking prescribed antihypertensive medications, and only 15.3% of patients had controlled hypertension [4]. The treatment of hypertension is facing great challenges. Actually, the low medication prices offer the possibility of good control for almost all hypertensive patients. Optimal medication compliance remains an important strategy for hypertension control [5], the effective treatment and blood pressure (BP) control contribute to a reduction in morbidity and mortality from CVD [6].

Suboptimal adherence to antihypertensive medication is a major contributor to poor BP control [7]. It has been reported that 10%–80% of the patients do not comply with antihypertensive drugs [8]. A Brazilian study reported that noncompliance related to underdosing was common [9]. Bernard Vrijens reported adherence to prescribed antihypertensive drug treatments by electronically compiled dosing histories [10]. One report also showed noncompliance rate was 46.2%, and only 43.9% were certain their BP was controlled [11]. Three-quarters of patients with hypertension did not reach an optimal BP level due to poor medication compliance [12]. Nonadherence was inversely related to age and male sex [13]. The risk of uncontrolled BP from poor compliance was 1.36 and 1.85 times higher compared with complete compliance [14]. Noncompliance to antihypertensive medications became the leading cause of poor control and thereby CVD and mortality worldwide [15]. Furthermore, Malaysia, Portugal, Cuba, and other regions experienced a significant increase in control rate among hypertension patients under the management of the primary health care [16–18]. To address the burden of hypertension, the Chinese government launched a hypertension management system under the Basic Public Health Service (BPHS) program in 2009. Primary care physicians are responsible for providing management services to hypertension patients. BPHS included free BP monitoring, hypertension medicine consultation, face-to-face follow-up health education, and lifestyle modification guidance, etc. [19] to improve compliance and BP control [20, 21]. The patients receiving BPHS management have indicated better compliance with antihypertensive medicine and control rate when compared with patients who did not receive the service [22, 23].

To the best of our knowledge, this differed from available confusion self-reported or questionnaires of antihypertensive medication adherence results [6, 7]. Furthermore, there is limited scientific literature on the relationship among different grades of medication compliance groups with BP control rate, CVD event, and survival probability in the meantime. This study innovatively combined the primary care physicians' face-to-face BP measurement and recorded the adherence of antihypertensive medication by individual case and aimed to highlight the importance of sustained high-grade compliance with antihypertensive medication. The result will contribute to the Chinese objective of a 60% BP control rate [24]. The experience of BPHS extensively used by primary care physicians for better antihypertensive medicine adherence will also largely benefit limited resource developing countries.

## 2. Methods

### 2.1. Data Sources and Study Participants

BPHS program in China requires primary care institutions to enroll patients 35 years of age and older who have been diagnosed with hypertension into the BPHS system and to provide them with hypertension care and management by primary care providers at no cost [19, 20]. The BPHS program is designed to provide registration, health records, health check-ups, follow-up visits, and medication guidance to hypertensive patients who are enrolled in the program by primary healthcare physicians. All patients' follow-up information is uploaded to the BPHS electronic information system in real time after continuous face-to-face follow-up by primary healthcare physicians. The data management system has strict quality control [25]. The baseline information questionnaire and the follow-up questionnaire can be found in Additional files 1 and 2.

In 2015, Fuwai Hospital of the Chinese Academy of Medical Sciences (CAMS) implemented the project “The China Hypertension Survey (CHS).” The specific sampling scheme and project implementation procedures have been published previously [4, 26]. In brief, we conducted a cross-sectional study in Yunnan Province to perform hypertension screening for 14,960 residents aged 18 years and older. After excluding those who were under 35 years of age, nonhypertensive, missing antihypertensive medication information, and unique identifiers, baseline information was collected on 3840 hypertensive patients aged 35 years and older with complete information.

**Figure 1:**
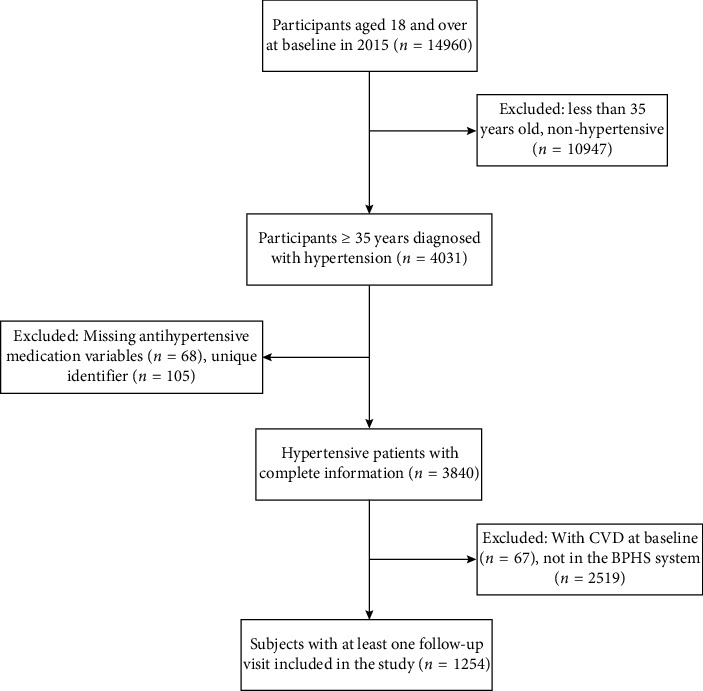
Flowchart of the participant selection process (abbreviation: CVD: cardiovascular disease; BPHS: basic public health service).

In addition, participants with prior CVD events at the baseline (*n* = 67) were excluded. As the BPHS electronic system in Yunnan Province was established in 2018, we established January 2018 as the first follow-up time. Linked to the BPHS e-system through the participants' unique identifiers, 1254 hypertensive patients registered in the BPHS system were included in this study. The inclusion process of participants is presented in Figure 1.

This study was approved by the Ethics Committee of Fuwai Hospital of CAMS (2012-402) and the Ethics Committee of Fuwai Yunnan Cardiovascular Hospital (2023-025-01). All respondents gave written informed consent according to BPHS requirements.

### 2.2. Data collection and measurement

Trained primary care physicians conducted questionnaires survey, including age, sex, ethnicity (Han vs. ethnic minority), educational level (elementary school and below, junior high school, and above), residence (urban vs. rural), smoking, alcohol drinking, CVD history, etc. After resting for five minutes, using a sphygmomanometer (OMRON HBP-1300, Kyoto, Japan) measured BP three times at 30 s intervals on the right arm. The three readings were averaged for analysis. The height was measured with a standard right-angle device and a fixed measurement tape (to the nearest 0.5 cm). Weight was measured by the OMRONV-BODYHBF-371 weighing scale (OMRON, Kyoto, Japan) to the nearest 0.1 kg.

According to the third edition of China BPHS Guidelines [20], trained primary care physicians also followed up and recorded each visiting BP measurement, self-reported CVD events, referral to higher hospitals for hypertensive emergencies, and death by the BPHS online version of “Follow-up Form of Hypertensive Patients.” In addition, “medication adherence” was assessed and recorded (regular medication, irregular medication) by the primary healthcare provider at each follow-up visit.

### 2.3. Definition of Variables

At the 5-year follow-up, each hypertensive patient may have different medication adherence at each stage, and we calculated the total number of regular medications taken over the 5 years and the total number of times each hypertensive patient was followed up. The percentage of medication compliance (range: 0%–100%) was calculated by numbers complying with the antihypertensive medicine over 5 years divided by the number of follow-ups over 5 years. According to the tertile method [27], < 87% of the follow-up subjects as the poor medication group, 87.0% and < 100% as the intermittent medication group, and 100% as the sustained medication group. This grouping description was also mentioned in our previous preprint [28].

Hypertension was defined as a previous history of hypertension diagnosis, or three consecutive BP measurements of systolic blood pressure (SBP) ≥ 140 mmHg or diastolic blood pressure (DBP) ≥ 90 mmHg, or ingestion of antihypertensive drugs in the past two weeks [29].

BMI was calculated as weight in kilograms divided by height in meters squared. We defined overweight as a BMI from 24.0 kg/m^2^ to less than 27.9 kg/m^2^ according to the Chinese adult body weight standard, and obesity as a BMI greater than or equal to 28 kg/m^2^ [30]. Current smoker was defined as at least 7 cigarettes per week for at least 6 months [31]. Participants who consumed at least one serving of alcoholic beverage per week in the past month were considered as current drinkers [32].

All follow-up data were obtained by the BPHS system. Through the BPHS database, we obtained all relevant information on individual names, identity cards, and addresses of hypertensive patients collected by all public healthcare units, and primary care physicians in Yunnan Province, China, followed up and filled out antihypertensive medications and dosages in an electronic format. CVD events, referrals due to severe hypertension or comorbidities, and deaths were recorded in the BPHS database.

### 2.4. Outcomes

The primary outcome was BP control, which was defined as SBP < 140 mmHg and DBP < 90 mmHg, while suboptimal or uncontrolled BP as either the SBP ≥ 140 mmHg or the DBP ≥ 90 mmHg [33].

Secondary outcomes were CVD, the occurrence of referrals, and death. CVD was defined as self-reported and doctor-confirmed (stroke or coronary heart disease), including angina pectoris (ICD-10 code I20), myocardial infarction (ICD-10 code I21-I24), heart failure (ICD-10 code I50), hemorrhagic stroke (ICD-10 code I60-I62), and ischemic stroke (ICD-10 code I63) [34–38]. All causes of death were registered by the primary health provider and confirmed by the local death registry, and referrals were defined as transfers to higher-level medical units.

### 2.5. Statistical Analyses

The mean and standard deviation were used to describe continuous variables, and categorical variables were expressed using frequency numbers (*n*) and percentages (%). The ANOVA or Kruskal–Wallis test was selected according to the distribution of variables to compare differences between different groups. Categorical data were analyzed using the chi-square test to compare differences between medication compliance groups.

We also used Pearson's correlation to show the difference between the follow-up last SBP/DBP and baseline SBP/DBP to analyze BP changes and used Spearman's rank correlation to show the relationship between 5-year cumulative follow-up times and changes in BP.

The Chordal graph was used to present the change of different antihypertensive compliance groups and BP control rates during the 5-year follow-up period, respectively.

Besides, we used a robust variance Poisson regression model to estimate the association between different medication groups and obtained incidence rate ratio (IRR) and 95% confidence intervals with the number of referrals after adjusting for the covariates at baseline (including age, residence, ethnicity, education, employment status, CVD, alcohol consumption, BMI, and baseline BP values).

Kaplan–Meier curves were used to calculate the cumulative risk of onset and survival probability of CVD events and compared with the log-rank test.

All statistical analyses were performed by R 4.0.5 and IBM SPSS 21.0. Statistical tests were 2-sided, and *p* < 0.05 was considered statistically significant.

## 3. Results

### 3.1. General Characteristics of the Participants

There were 1254 baseline-targeted hypertension patients in 2015. The average age was 65.6 ± 11.1 years, 547 (43.6%) were male, 451 (36.0%) living in urban areas, 746 (59.5%) were Han ethnicity, 1027 (81.9%) with primary school education and below, and 796 (63.5%) currently employed. The antihypertensive medications taken over the 5 years are shown in Additional file 3.

Of the three antihypertension medicine compliance groups, 421 (33.6%) were found in the poor group, 192 (15.3%) in the intermittent group, and 641 (51.1%) in the sustained group. The mean age of the poor compliance group was higher. Intermittent and sustained compliance groups had longer durations of hypertension and were more frequently overweight or obese. Fewer of the intermittent or sustained compliers were current drinkers, see Table 1.

The baseline SBP was higher in the poor compliance group (157.07 ± 19.37 mmHg) than the intermittent group (155.55 ± 21.17 mmHg) and the sustained group (152.88 ± 21.64 mmHg). DBP was similar in the poor (84.86 ± 12.82 mmHg), intermittent (85.92 ± 12.91 mmHg), and sustained compliance (83.01 ± 12.66 mmHg) groups. The 992, 1218, 1121, 1066, and 999 patients, respectively, were followed up every year for 5 years from 2018 to 2022, see Table 1.

### 3.2. Blood Pressure Difference in the Three Compliance Groups

**Figure 2:**
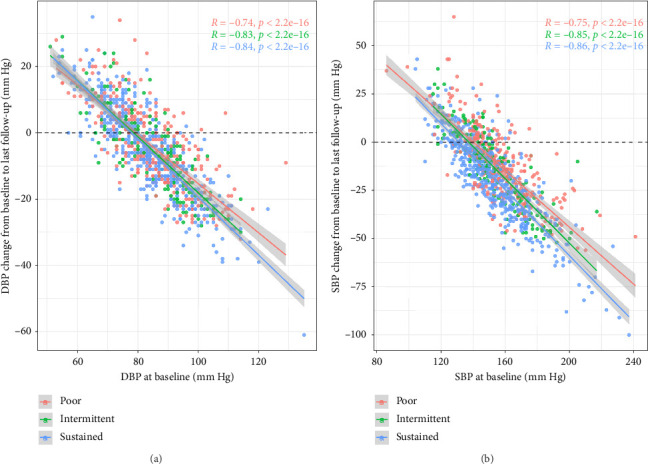
Relationship of different antihypertensive medicine compliance groups and systolic (b) and diastolic pressure (a) changes from 2015 to 2022.

Figure 2(a) shows the change in DBP between 2015 and the last follow-up as colored points for each of the patients. The three colors represent the three compliance groups. Calculated linear regression lines for the three groups are also shown in Yunnan Province 2 and calculated correlation coefficients are displayed. Decreasing tendency is most marked for the sustained group.

Figure 2(b) shows the change in SBP between the baseline and last follow-up plotted against baseline systolic pressure. The same color scheme is used and regression lines are displayed. Compared with the baseline in 2015, SBP decreased in all three groups in 2022, with the largest decrease in the sustained group (2015: 152.88 ± 21.64 mmHg vs. 2022: 134.61 ± 10.49 mmHg, *p* < 0.05) and the least decrease in the poor group (2015: 157.07 ± 15.37 mmHg vs. 2022: 140.33 ± 12.04 mmHg, *p* < 0.05). The difference in the relationships between the baseline and change in SBP is more marked than for DBP.

### 3.3. BP Difference in Three Compliance Groups With Cumulative Follow-Up Times

**Figure 3:**
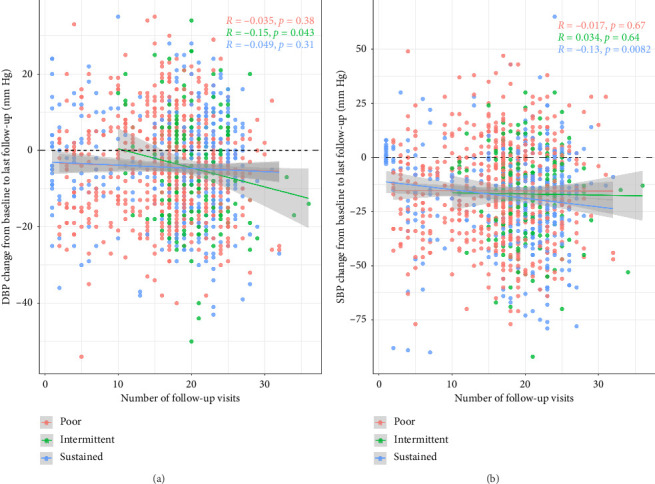
Relationship between the follow-up times and the change of systolic (b) and diastolic pressure (a) from 2015 to 2022.

Figure 3 is a scatter plot of change from baseline in diastolic and SBP for each participant at each follow-up visit. The points are color coded for the three compliance groups. Linear regression lines are also displayed. During the 5-year follow-up, all three groups showed different degrees of systolic and diastolic pressure decrease, and as the cumulative number of times of follow-up showed DBP from the intermittent group (detail for Figure 3(a)), systolic pressure from the sustained group (detail for Figure 3(b)) decreased remarkably (*p* < 0.05), a moderate decrease of DBP from poor, and SBP from poor and intermittent group tend to level off (*p* > 0.05).

### 3.4. BP Control Rate in Three Compliance Groups

**Figure 4:**
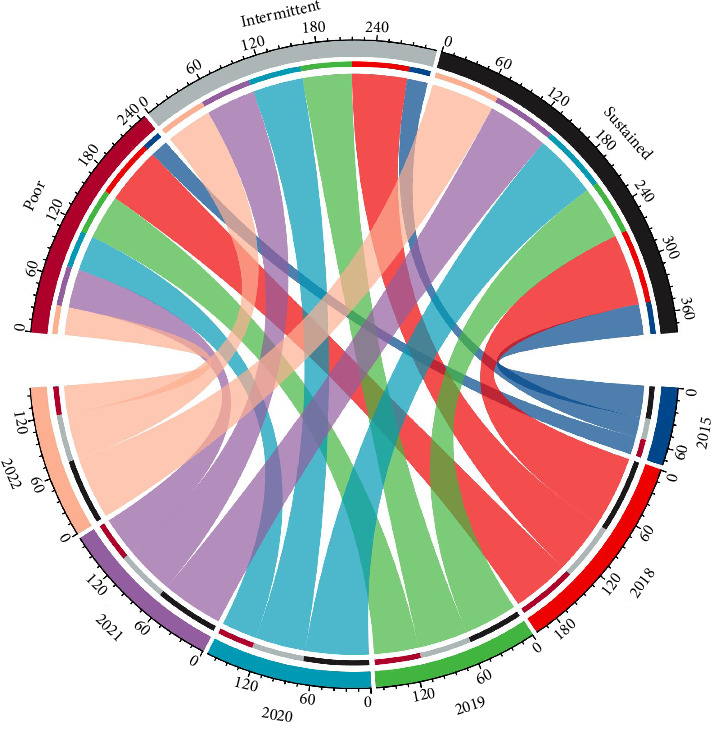
Hypertension control rates from poor, intermittent, and sustained groups between 2015 and 2022.

In 2015, control rates in the poor, intermittent, and sustained compliance groups were 19.7%, 22.5%, and 34.4%, respectively. After the initiation of treatment by the primary care physician from the BPHS, compliance rates increased to 63.0%, 61.0%, and 79.0% in 2018, 49.4%, 54.8%, and 59.0% in 2019, 39.2%, 55.5%, and 70.3% in 2020, 42.5%, 54.5%, and 67.9% in 2021, and 30.5%, 50.8%, and 70% in 2022, respectively, see Figure 4 and Additional file 4.

### 3.5. Referrals in the Three Compliance Groups

Table 2 shows the relationships between patient referrals for unsatisfactory BP control and compliance group using the sustained group as a reference. The number of referrals due to related complications was 11.5% higher in the adverse group than in the sustained group (IRR = 1.115 and 95% CI: 1.043–1.193) and 10.5% higher in the intermittent group (IRR = 1.105 and 95% CI: 1.011–1.206).

### 3.6. The Probability of CVD Event in Three Compliance Groups

**Figure 5:**
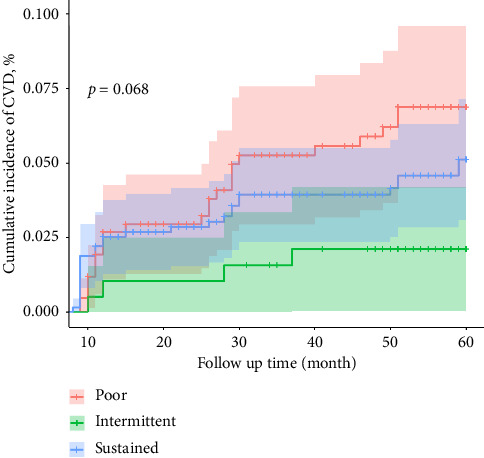
Probability of CVD events from 3 antihypertension medicine groups, 2018–2022.

Although CVD (cardiovascular and stroke) occurred in all three groups, the probability of occurrence was not high in 5 years (60 months), all below 7.5%. However, the highest probability of CVD occurrence came from the poor group, see Figure 5. For other comorbidities, see Additional file 5.

### 3.7. Survival Probability in Three Compliance Groups

**Figure 6:**
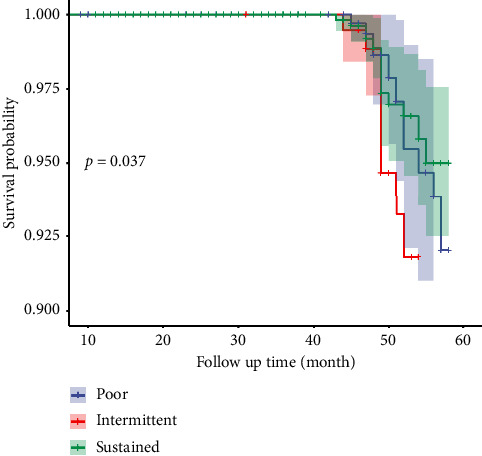
Survival curves from 3 compliance groups, 2018–2022.

After 5 years (60 months) of follow-up, the survival probability in the sustained group was the highest and kept at 0.950 at the end of 5 years, which was significantly higher than the poor and intermittent groups (*p* < 0.05). See Figure 6.

## 4. Discussion

This study has provided several more reliable and important insights into the relationships between different grades of antihypertension compliance to BP decreasing, BP control rate, preventing cardiovascular events and different survival probability over a 5-year period, as the results directly recorded by BPHS management.

This longitudinal study focused on 1254 hypertension patients from a baseline survey in 2015 with 5 years follow-up from 2018 to 2022. SBP declined from 154.69 ± 20.90 mmHg to 137.48 ± 12.44 mmHg and DBP from 84.08 ± 12.80 mmHg to 79.50 ± 7.88 mmHg. Patients with higher baseline values had a more pronounced decrease in BP. According to a previous study [39], every 10 mmHg lower BP can reduce about 30% of the risk of stroke. A survey of 1.7 million adults in China showed that poor BP control resulted in 24,914,000 years of life lost and 28,657 million life quality years lost [40]. Under the management of the BPHS project, the BP control rate of three antihypertensive medicine compliance groups reached 70% in 2022, significantly higher than 34.4% at the baseline, and the intermittent group and poor group also increased from 22.5% to 19.7% at the baseline to 50.8% and 30.5%, respectively. Consistent with the reports of primary care is associated with patient hypertension medication adherence and BP control [41]. Therefore, there is an urgent need to ensure the accessibility and coverage of BPHS to help people with hypertension effectively control their BP. Our study results showed the lowest control rate in the poor compliance group and the highest in the sustained group. The control rate in 2022 in the poor group was less than half of that in the sustained group. Primary care physicians offered BP measurement, medication guidance, and related lifestyle modification guidance [19], thus improves the patient's awareness of hypertension,\ so that they pay more attention to their condition. Adherence to antihypertensive drugs is undoubtedly a very effective strategy to reduce BP and prevent CVD events [42].

Our results revealed that even under the premise of taking antihypertensive drugs if hypertension patients do not comply with medication regimens, the BP control rate under BPHS gradually decreases over time. The control rate of the poor compliance group decreased year by year (63%–49.4%–39.2%–42.5%–30.5%). Noncompliance to medication means that the patient's medication behavior is inconsistent with the doctor's advice, which prevents the full benefit of the prescribed treatment, and results in early complications [43]. Hypertension requires often lifelong treatment, persistence is very important for its management [44]. However, adherence to antihypertensive treatment has been the lack of direct, sufficient methods [13] and a persistent gap remains between stated public health targets and achieved BP control rates [45]. Our research analyzed different grades of antihypertension compliance results over 5 years from the formatted online recorded data by primary care physicians. Our results showed the importance of long-term sustained medication compliance for sustained BP control, furthermore following the strategies for improving adherence to antihypertensive medications at both the individual and health [45].

This study also found that the risk of referrals due to related complications in the poor compliance group and the intermittent group was higher than in the sustained group. The sustained group with sustained medication adherence had a much higher survival probability than the poor compliance group. From a public health perspective, hypertension management has enormous health and socioeconomic benefits [46]. Other research [47, 48] shows that a reduction of SBP by 10 mmHg or DBP by 5 mmHg reduces CVD risk. Improved compliance saves billions in direct healthcare costs and indirect losses from reduced economic productivity [49].

This study explored the individual hypertension patient BP and medicine regularly recorded by primary care physicians face to face, the activity strengthened better long-term antihypertensive medication adherence from both health provider the individual patient sides, consistent with the World Health Organization report “Adherence to Long-Term Therapy: Evidence for Action” [50]. The study contributes to the UN sustainable development goal of lowering chronic illness fatalities by one-third by 2030 [51]. The face-to-face measure of the BP and recording the adherence to antihypertensive medication by primary care physicians provide more reliable results. The 5-year follow-up of three adherence grades to the antihypertensive medicine and the connection between adherence to BP control, CVD events, and mortality is more stable.

There are some limitations to this study. First, from 2016 to 2017, the follow-up results were not collected, forming a gap of two-year results and a loss of some patients. Second, this study did not reflect other unmeasured factors affecting medication compliance such as patients-centered primary care, particularly providers' communication skills and antihypertensive medication adherence behavior [41], etc. Third, the primary care physicians may not accurately determine medication compliance, but we used 5 years of longitudinal data to analyze medication adherence to minimize this potential bias. Future follow-up studies may require a larger sample size to improve representativeness and extrapolation.

## 5. Conclusions

The study indicates that hypertensive patients' long-term compliance with antihypertensive drugs can significantly reduce systolic and DBP, improve BP control rate, increase survival probability, and reduce the risk of CVD events. Besides, the BPHS program can greatly promote consistent and medication adherence for hypertensive patients. The study also provides a potential perspective for resource limited developing countries that are the connection of primary care providers with adherence to the antihypertensive medication by electronic data system.

## Figures and Tables

**Table 1:** Descriptive characteristics of baseline survey of 1254 hypertension patients according to the three groups in 2015.

Characteristics	Total	Poor	Intermittent	Sustained	*p*
Total, *n* (%)	1254 (100.0)	421 (33.6)	192 (15.3)	641 (51.1)	
Age (y)	65.6 ± 11.1	66.01 ± 11.42	63.58 ± 11.00	65.96 ± 10.91	0.023
< 65	541 (43.1)	172 (40.9)	94 (49.0)	275 (42.9)	0.075
65–74	408 (32.5)	134 (31.8)	66 (34.4)	208 (32.4)	
≥ 75	305 (24.3)	115 (27.3)	32 (16.7)	158 (24.6)	
Male sex	547 (43.6)	192 (45.6)	90 (46.9)	265 (41.3)	0.240
Urban residence	451 (36.0)	110 (26.2)	62 (32.3)	279 (43.5)	< 0.001
Han ethnicity	746 (59.5)	239 (56.8)	100 (52.1)	407 (63.5)	0.007
Education, elementary school, and below	1027 (81.9)	363 (86.2)	157 (81.8)	507 (79.1)	0.013
Occupation, employed	796 (63.5)	257 (61.0)	133 (69.3)	406 (63.3)	0.145
Duration of hypertension (y)					
< 5	809 (64.5)	297 (70.5)	127 (66.1)	385 (60.1)	0.005
5–10	321 (25.6)	96 (22.8)	47 (24.5)	178 (27.8)	
> 10	124 (9.9)	28 (6.7)	18 (9.4)	78 (12.2)	
Body mass index (kg/m^2^)	24.36 ± 3.80	23.84 ± 3.69	24.38 ± 3.80	24.69 ± 3.82	0.002
Overweight or obesity	612 (48.8)	185 (43.9)	98 (51.0)	359 (56.0)	0.001
Current smoker	308 (24.6)	111 (26.4)	51 (26.6)	146 (22.8)	0.324
Current drinker	416 (33.2)	170 (40.4)	67 (34.9)	179 (27.9)	< 0.001
SBP (mmHg)	154.69 ± 20.90	157.07 ± 19.37	155.55 ± 21.17	152.88 ± 21.64	0.005
DBP (mmHg)	84.08 ± 12.80	84.86 ± 12.82	85.92 ± 12.91	83.01 ± 12.66	0.007
Newly diagnoses patients	416 (33.2)	172 (41.3)	54 (13.0)	190 (45.7)	< 0.001
History diagnosed patients	838 (66.8)	249 (29.7)	138 (16.5)	451 (53.8)	

Abbreviations: DBP, diastolic blood pressure; SBP, systolic blood pressure.

**Table 2:** Poisson regression models for the number of referral and associated variables among hypertensive patients.

Variables		IRR	95% CI	*p*
Group	Poor	1.115	(1.043–1.193)	0.001
	Intermittent	1.105	(1.011–1.206)	0.026
	Sustained	1^†^		

Residence	Urban	0.703	(0.659–0.751)	< 0.001
	Village	1^†^		

Occupation	Employed	0.458	(0.424–0.493)	< 0.001
	Unemployed and other	1^†^		

Education	Junior high school and above	0.809	(0.748–0.873)	< 0.001
	Primary and below	1^†^		

Ethnic	Non-Han	0.817	(0.767–0.869)	< 0.001
	Han	1^†^		

^†^Reference.

## Data Availability

The data supporting the findings of this study and its Supporting Information are available on request from the corresponding author.

## Supporting Information

Supporting Information 1 Additional File 1: Baseline survey questionnaire.

Supporting Information 2 Additional File 2: Basic Public Health Services Management of hypertensive patients' follow-up questionnaire.

Supporting Information 3 Additional File 3: Patients on different antihypertensive drugs during the 5-year follow-up period.

Supporting Information 4 Additional File 4: BP control rates at baseline and 5-year follow-up.

Supporting Information 5 Additional File 5: Comorbidities in patients taking different antihypertensive drugs at 5-year follow-up.
