# Supplementary material for: Long-Term Effects of Sustained Regular Medication in Hypertensive Patients in Yunnan, China: A Cohort Study of 5 Years' Follow-Up
Source: Int J Hypertens. 2025 May 8;2025:4505824. doi: 10.1155/ijhy/4505824 (PMC12081157; doi:10.1155/ijhy/4505824)
Supplement: Supporting Information 1 — Additional File 1: Baseline survey questionnaire. [file 4505824.f1.docx]

Informed Consent Form

(For survey participants aged 18 and above)

Entrusted by the Ministry of Science and Technology and the Ministry of Health of the People's Republic of China, the National Center for Cardiovascular Diseases, in collaboration with relevant national departments, is conducting the "Prevalence Survey and Key Technology Research of Major Cardiovascular Diseases in China" as part of a national science and technology support program. The aim of this survey is to understand the prevalence of cardiovascular diseases and the levels of risk factors among the Chinese population. During this survey, we will ask about your personal and family general information, physical activity, lifestyle and behaviors, and medical history. We will also measure your height, weight, body fat, and blood pressure. These tests will help you understand your own health status. If conditions permit in the future, we may also conduct follow - up on your health status over a certain period.

All relevant examinations will be carried out with your consent, and all examination fees are waived. After the completion of all examination items, we will feedback the examination results to you.

The data obtained from this survey will only be used for disease prevention and treatment research, providing a basis for the country to formulate disease control policies and medical reform plans. All your personal information and examination results will be strictly confidential and will never be disclosed or made public.

Before signing this consent form, I have read or had it read to me, and I have had sufficient opportunity to ask questions and received satisfactory answers. I agree to participate in this survey.

Signature of the survey participant Or signature of the proxy:

Date of signature:

I confirm that I have introduced the details of this project to the survey participant.

Signature of the investigator:

Date of signature:

Basic Information

A1

Province:

City:

District/County:

Residential/Village Committee:

Number:

A2

Name:

A3

Gender:

1 = Male, 2 = Female

A4

Date of birth:

A5

ID number:

A6

Work unit (or village) and department:

A7

Detailed home address:

A8

Telephone number:

Home:

(Office):

(Mobile):

A9

Contact person's name:

Relationship with the participant:

A11

Contact person's work unit:

A12

Contact person's address:

A13

Contact person's telephone number:

Home:

(Office):

(Mobile):

A14

Ethnic group:

1 = Han, 2 = Mongolian, 3 = Hui, 4 = Tibetan, 5 = Uyghur, 6 = Miao, 7 = Yi, 8 = Zhuang, 9 = Bouyei, 10 = Korean, 11 = Manchu, 12 = Dong, 13 = Yao, 14 = Bai, 15 = Tujia, 16 = Hani, 17 = Kazak, 18 = Dai, 19 = Li, 20 = Other ethnic groups (please specify)

A15

Educational level:

1 = No schooling, 2 = Primary school, 3 = Junior high school, 4 = Senior high school/Technical secondary school, 5 = University, 6 = Postgraduate

A16

Marital status:

0 = Unmarried, 1 = Married/Remarried/Cohabiting, 2 = Separated, 3 = Divorced, 4 = Widowed

A17

Medical security systems enjoyed: (Multiple choices, fill in 1 if applicable, 0 if not)

1 = Urban Employee Medical Insurance □ 2 = New Rural Cooperative Medical Scheme □ 3 = Urban Resident Medical Insurance □

4 = Commercial Medical Insurance □ 5 = Free Medical Care □ 6 = Others

A18

Employment status:

1 = Employed, 2 = Retired, 3 = Student, 4 = Unemployed or job - seeking

A19

Current occupation (including the current occupation of rehired persons):

1 = Managers of government agencies, enterprises, and public institutions, 2 = Professional and technical personnel, 3 = General office workers and related personnel, 4 = Commercial and service industry personnel, 5 = Self - employed individuals, 6 = Non - agricultural industrial workers,

7 = Farmers engaged in non - agricultural labor, 8 = Agricultural laborers (engaged in agriculture, forestry, animal husbandry, and fishery), 9 = Others

A19.1

If retired, occupation before retirement (classified as above, fill in 0 for those without the concept of retirement such as farmers and those not retired):

A20

Are you a local registered resident? 0 = No, 1 = Yes

A21

Length of residence in the local area: 1 = 0 - 6 months, 2 = 7 - 12 months, 3 = 12 - 36 months, 4 = 36 months or more

National Center for Cardiovascular Diseases, Fuwai Hospital, Chinese Academy of Medical Sciences

Version: 201200803

Health Status

Part 1: Lifestyle and Behaviors

I. Smoking (including tobacco leaves and cigarettes)

B1.1

What was your smoking situation in the past 30 days? 0 = Do not smoke (skip to B1.3), 1 = Smoke every day, 2 = Smoke, but not every day

B1.1.1

How long have you had the habit of smoking every day? 1 = < 3 months, 2 = 3 - 6 months, 3 = 6 - 12 months, 4 = More than 1 year (____ years)

B1.2

What is your usual smoking amount? (Investigator note: Fill in "999 or 999.9" if unsure)

Smoking amount:

B1.2.1

Manufactured cigarettes (pieces):

B1.2.2

Hand - rolled cigarettes (tael):

B1.2.3

Shag/tobacco pipe (tael):

B1.2.4

Cigars (pieces):

B1.2.5

Others: ________ (unit: )

Smoking frequency*:

0 = Do not smoke, 1 = Daily, 2 = Weekly, 3 = Monthly (skip to B1.4)

B1.3

Did you smoke in the past?

0 = Do not smoke (skip to B1.8), 1 = Smoke every day, 2 = Smoke, but not every day

B1.4

At what age did you start smoking?

B1.5

How many years have you smoked in total?

B1.6

What is the total amount of cigarettes you have smoked from the first time you were exposed to tobacco until now? 1 = < 20 pieces (1 pack of cigarettes), 2 = 20 - 400 pieces (20 packs of cigarettes), 3 = More than 400 pieces

B1.7

Have you ever quit smoking (meaning you seriously considered quitting and took action)? 0 = No, 1 = Yes

B1.7.1

How many years have you quit smoking in total?

B1.7.2

Why did you quit smoking? 1 = Illness, 2 = Others

B1.8

Under normal circumstances, how many days a week do you expose to second - hand smoke in an enclosed environment? (This question is not asked for current smokers) 0 = Almost none (skip to II. Drinking), 1 = Yes, for _____ days

B1.8.1

How many days have you been exposed for more than 15 minutes in total? 0 = None (skip to II. Drinking), 1 = Yes, for _____ days

National Center for Cardiovascular Diseases, Fuwai Hospital, Chinese Academy of Medical Sciences

Version: 201200803

II. Drinking

B2.1

Have you ever drunk alcohol? 0 = No (skip to III. Diet), 1 = Yes

B2.2

At what age did you start drinking regularly? _____ years old

B2.3

What kind of drinking habit do you have? 1 = At least once a day, 2 = At least once a week, 3 = At least once a month, 4 = Seasonal drinking, drinking for ____ months a year, at least ____ times a month, 5 = Occasional drinking, drinking ____ times a year

B2.4

In the past month, have you drunk alcohol at least once a week? 0 = No, 1 = Yes

B2.5

Do you usually drink until you're drunk?

1 = Almost every time, 2 = Most of the time, 3 = Half of the time, 4 = Less often, 5 = Rarely, 6 = Never

B2.6

Have you ever quit drinking? 0 = No, 1 = Yes

B2.6.1

At what age did you start quitting drinking? _____ years old

B2.6.2

How many years have you quit drinking in total? _____ years

B2.6.3

Why did you quit drinking? 1 = Illness, 2 = Others:

B2.7

What types of alcohol do you drink and what is your drinking amount? (Current drinkers fill in the current situation, non - current drinkers fill in the past situation):

Average amount per drink:

Months/Year:

Alcohol content:

B2.7.1 Drinking frequency*:

0 = Do not drink, 1 = Daily, 2 = Weekly, 3 = Monthly, 4 = Yearly.

III. Diet (to understand the frequency and amount of the following foods you consumed in the past year)

B3.1 Food

Consumption frequency (choose one)

Consumption amount

B3.1.1 Rice, flour, coarse grains (millet, sorghum, corn, etc.)

0 = Do not eat, 1 = Daily, 2 = Weekly, 3 = Monthly, 4 = Yearly

□ jin □ tael

B3.1.2 Tubers (sweet potatoes, yams, taros, potatoes, etc.)

0 = Do not eat, 1 = Daily, 2 = Weekly, 3 = Monthly, 4 = Yearly □

□ jin □ tael

B3.1.3 Fresh vegetables (excluding dried vegetables and pickled vegetables)

0 = Do not eat, 1 = Daily, 2 = Weekly, 3 = Monthly, 4 = Yearly □

□ jin □ tael

B3.1.4 Livestock meat (pork, beef, mutton, etc.)

0 = Do not eat, 1 = Daily, 2 = Weekly, 3 = Monthly, 4 = Yearly □

□ jin □ tael

B3.1.5 Poultry meat (chicken, duck, goose, etc.)

0 = Do not eat, 1 = Daily, 2 = Weekly, 3 = Monthly, 4 = Yearly □

□ jin □ tael

B3.1.6 Fish

0 = Do not eat, 1 = Daily, 2 = Weekly, 3 = Monthly, 4 = Yearly □

□ jin □ tael

B3.1.7 Shrimp, crab, shellfish and other aquatic products

0 = Do not eat, 1 = Daily, 2 = Weekly, 3 = Monthly, 4 = Yearly □

□ jin □ tael

B3.1.8 Eggs (chicken eggs, duck eggs, etc.)

0 = Do not eat, 1 = Daily, 2 = Weekly, 3 = Monthly, 4 = Yearly □

□ jin □ tael

B3.1.9 Dairy products (converted to fresh milk)

0 = Do not eat, 1 = Daily, 2 = Weekly, 3 = Monthly, 4 = Yearly □

□ jin □ tael

B3.1.10 Soy products (calculated as tofu)

0 = Do not eat, 1 = Daily, 2 = Weekly, 3 = Monthly, 4 = Yearly □

□ jin □ tael

B3.1.11 Fresh fruits

0 = Do not eat, 1 = Daily, 2 = Weekly, 3 = Monthly, 4 = Yearly □

□ jin □ tael

B3.1.12 Dried fruits (peanuts, melon seeds, walnuts)

0 = Do not eat, 1 = Daily, 2 = Weekly, 3 = Monthly, 4 = Yearly □

□ jin □ tael

B3.1.13 Pickled vegetables/pickled cabbage/preserved vegetables

0 = Do not eat, 1 = Daily, 2 = Weekly, 3 = Monthly, 4 = Yearly □

□ jin □ tael

B3.2

How many kilogram of vegetable oil does your family usually consume per month?

_____ kilogram/month

B3.3

How many kilogram of animal oil does your family usually consume per month?

_____ kilogram/month

B3.7

How many kilogramof salt does your family usually consume per month?

_____ kilogram/month

IV. Physical Activity

The following questions are about your various physical activities (including agricultural work, work, housework, transportation - related physical activities, and recreational exercises or sports) in a typical week.

B4.1 Work, agricultural, and housework - related physical activities

B4.1.1 During your work, agricultural work, and housework, do you have high - intensity activities that last for more than 10 minutes? (High - intensity activities refer to activities that require significant physical effort, such as lifting heavy objects or digging, or that cause a significant increase in breathing and heart rate) Investigator note: You can show the physical activity classification table.

1 = Yes, 2 = No

B4.1.2 In a typical week, how many days do you engage in the above - mentioned high - intensity activities during your work, agricultural work, and housework?

（ ） days

B4.1.3 In a typical day, how long do you engage in the above - mentioned high - intensity activities in total during your work, agricultural work, and housework? (Do not count activities that last less than 10 minutes each time)

（ ） hours （ ） minutes

￼

B4.1.4 During your work, agricultural work, and housework, do you have medium - intensity activities that last for more than 10 minutes? (Medium - intensity activities refer to activities that require moderate physical effort, such as sawing wood, doing laundry, or cleaning, or that cause a mild increase in breathing and heart rate) Investigator note: You can show the physical activity classification table.

1 = Yes, 2 = No

B4.1.5 In a typical week, how many days do you engage in the above - mentioned
