# Supplementary material for: Long-Term Effects of Sustained Regular Medication in Hypertensive Patients in Yunnan, China: A Cohort Study of 5 Years' Follow-Up
Source: Int J Hypertens. 2025 May 8;2025:4505824. doi: 10.1155/ijhy/4505824 (PMC12081157; doi:10.1155/ijhy/4505824)
Supplement: Supporting Information 2 — Additional File 2: Basic Public Health Services Management of hypertensive patients' follow-up questionnaire. [file 4505824.f2.docx]

**Follow-up Record of Hypertensive Patients in the National Basic Public Health Service Project**

Name：

ID Number ：

Follow-up Date：

Newly Developed Complications：

Please select the diseases or conditions that the patient has newly developed since the last follow-up (multiple choices are available):

□ None □ Newly developed ischemic stroke, Onset Date：

□ Newly developed angina pectoris, Onset Date：

□ Newly developed myocardial infarction, Onset Date

□ Newly developed hemorrhagic stroke, Onset Date

□ Newly developed stroke, unspecified, Onset Date

□ After coronary intervention (stent) surgery, Onset Date

□ Diabetes, Onset Date

□ After coronary artery bypass grafting, Onset Date

□ Chronic kidney disease, Onset Date

Physical Examination

□ Newly developed heart failure, Onset Date

□ Peripheral vascular disease, Onset Date

□ Atrial fibrillation, Onset Date

Blood Pressure： (mmHg)

Heart Rate： (beats/minute)

Height (cm)：

Weight (kg)：

Waist Circumference： (cm)

**Smoking statue**

1.Are you currently smoking?

□ Yes, smoking almost every day or most days.

□ Yes, smoking occasionally.

□ No

1. At what age did you start the habit of smoking every day or almost every day? - years old.
2. If you choose "Yes", did you smoke in the last 7 days?

□ Yes □ No, never smoked. □ No, have quit smoking.

**Auxiliary Examinations**

Please select the examinations done since the last outpatient visit or those to be done during this outpatient visit (multiple choices are available).

□ None □ Blood routine □ Urine routine □ Blood electrolytes (potassium, sodium, nitrogen) □ Blood creatinine □ Blood glucose □ Blood lipids

□ Electrocardiogram

**Current Medications and Compliance**

Please confirm the antihypertensive, lipid-lowering, or antiplatelet medications the patient is currently taking. You can add to the following table. Record the compliance of antihypertensive medications taken since the last follow-up:

Drug Name：

Single Dose and Unit ：

Frequency of Medication：

Administration Route：

Compliance of Antihypertensive Medication：

Reasons for Non-compliance ：

**Diagnosis:**

□ Hypertension □ Diabetes □ Hyperlipidemia □ Coronary heart disease □ Angina pectoris □ Myocardial infarction □ After coronary intervention (stent) surgery □ After coronary artery bypass grafting □ Heart failure □ Atrial fibrillation □ Stroke, unspecified □ Ischemic stroke □ Hemorrhagic stroke □ Chronic kidney disease □ Chronic obstructive pulmonary disease □ Bronchial asthma □ Gout □ Peripheral vascular disease □ Others

**Drug Treatment**

The drug treatment plan after this visit (only record antihypertensive, lipid-lowering, and antiplatelet drugs. Add or delete from the current medication list):

Single Dose and Unit ：

Frequency of Medication：

Administration Route：

Is Referral Needed?

□ Yes □ No

Referral Dat：

Reasons for Referral ：

Referral Hospital：

Follower-up Staff /Inputter:

Next Follow-up Date :
