# Supplementary material for: Long-Term Effects of Sustained Regular Medication in Hypertensive Patients in Yunnan, China: A Cohort Study of 5 Years' Follow-Up
Source: Int J Hypertens. 2025 May 8;2025:4505824. doi: 10.1155/ijhy/4505824 (PMC12081157; doi:10.1155/ijhy/4505824)
Supplement: Supporting Information 3 — Additional File 3: Patients on different antihypertensive drugs during the 5-year follow-up period. [file 4505824.f3.docx]

Additional file 3: Patients on different antihypertensive drugs during the 5-year follow-up period

| Year | **Poor** | **Intermittent** | **Sustained** | ***$\chi^{2}$*** | ***p*-Value** |
| --- | --- | --- | --- | --- | --- |
| 2018 |  |  |  | 49.664 | <0.001 |
| Not taking antihypertensive drugs | 153(36.3) | 70(36.5) | 240(37.4) |  |  |
| ACEIs | 51(12.1)^c^ | 21(10.9)^c^ | 29(4.5)^ab^ |  |  |
| ARBs | 10(2.4) | 2(1.0) | 23(3.6) |  |  |
| beta-blockers | 8(1.9) | 4(2.1) | 15(2.3) |  |  |
| CCBs | 112(26.6)^c^ | 64(33.3) | 215(33.5)^a^ |  |  |
| diuretics | 66(15.7)^c^ | 23(12.0) | 57(8.9)^a^ |  |  |
| Other | 21(5.0) | 8(4.2)^c^ | 62(9.7)^b^ |  |  |
| 2019 |  |  |  | 67.249 | <0.001 |
| Not taking antihypertensive drugs | 98(23.5)^bc^ | 15(7.8)^ac^ | 106(16.5)^ab^ |  |  |
| ACEIs | 55(13.1) | 23(12.0)^c^ | 41(6.4)^b^ |  |  |
| ARBs | 11(2.6)^c^ | 5(2.6) | 40(6.2)^a^ |  |  |
| beta-blockers | 7(1.7) | 6(3.1) | 22(3.4) |  |  |
| CCBs | 150(35.6)^bc^ | 95(49.5)^a^ | 280(43.7)^a^ |  |  |
| diuretics | 76(18.1)^c^ | 32(16.7) | 80(12.5)^a^ |  |  |
| Other | 23(5.5)^c^ | 16(8.3) | 72(11.2)^a^ |  |  |
| 2020 |  |  |  | 57.333 | <0.001 |
| Not taking antihypertensive drugs | 112(26.6)^bc^ | 15(7.8)^ac^ | 127(19.8)^ab^ |  |  |
| ACEIs | 45(10.7) | 20(10.4) | 43(6.7) |  |  |
| ARBs | 9(2.1)^c^ | 6(3.1) | 40(6.2)^a^ |  |  |
| beta-blockers | 6(1.4) | 7(3.6) | 23(3.6) |  |  |
| CCBs | 151(35.9)^b^ | 98(51.0)^a^ | 274(42.7) |  |  |
| diuretics | 72(17.1) | 33(17.2) | 81(12.6) |  |  |
| Other | 26(6.2) | 13(6.8) | 53(8.3) |  |  |
| 2021 |  |  |  | 69.959 | <0.001 |
| Not taking antihypertensive drugs | 125(29.7)^b^ | 16(8.3)^ac^ | 155(24.2)^b^ |  |  |
| ACEIs | 43(10.2) | 20(10.4)^c^ | 35(5.5)^b^ |  |  |
| ARBs | 8(1.9)^c^ | 4(2.1) | 39(6.1)^a^ |  |  |
| beta-blockers | 3(0.7)^b^ | 7(3.6)^a^ | 15(2.3) |  |  |
| CCBs | 145(34.4)^bc^ | 99(51.6)^a^ | 276(43.1)^a^ |  |  |
| diuretics | 70(16.6) | 32(16.7) | 76(11.9) |  |  |
| Other | 27(6.4) | 14(7.3) | 45(7.0) |  |  |
| 2022 |  |  |  | 42.482 | <0.001 |
| Not taking antihypertensive drugs | 142(33.7)^b^ | 28(14.6)^ac^ | 192(30.0)^b^ |  |  |
| ACEIs | 37(8.8) | 18(9.4) | 37(5.8) |  |  |
| ARBs | 16(3.8) | 6(3.1) | 35(5.5) |  |  |
| beta-blockers | 3(0.7)^b^ | 9(4.7)^a^ | 16(2.5) |  |  |
| CCBs | 149(35.4)^b^ | 94(49.0)^a^ | 254(39.6) |  |  |
| diuretics | 46(10.9) | 25(13.0) | 60(9.4) |  |  |
| Other | 28(6.7) | 12(6.3) | 47(7.3) |  |  |

Note: a comparison with the poor group; b comparison with the Intermitten**t** group; c comparison with the Sustained
