# Supplementary material for: Long-Term Effects of Sustained Regular Medication in Hypertensive Patients in Yunnan, China: A Cohort Study of 5 Years' Follow-Up
Source: Int J Hypertens. 2025 May 8;2025:4505824. doi: 10.1155/ijhy/4505824 (PMC12081157; doi:10.1155/ijhy/4505824)
Supplement: Supporting Information 4 — Additional File 4: BP control rates at baseline and 5-year follow-up. [file 4505824.f4.docx]

Additional file 4 Blood pressure control rates at baseline and 5 - year follow-up

| Year of follow-up | Poor | Intermittent | Sustained |
| --- | --- | --- | --- |
| 2015 | 19.7 | 22.5 | 34.4 |
| 2018 | 63 | 61 | 79 |
| 2019 | 49.4 | 54.8 | 59 |
| 2020 | 39.2 | 55.5 | 70.3 |
| 2021 | 42.5 | 54.5 | 67.9 |
| 2022 | 30.5 | 50.8 | 70 |
