# Supplementary material for: Long-Term Effects of Sustained Regular Medication in Hypertensive Patients in Yunnan, China: A Cohort Study of 5 Years' Follow-Up
Source: Int J Hypertens. 2025 May 8;2025:4505824. doi: 10.1155/ijhy/4505824 (PMC12081157; doi:10.1155/ijhy/4505824)
Supplement: Supporting Information 5 — Additional File 5: Comorbidities in patients taking different antihypertensive drugs at 5-year follow-up. [file 4505824.f5.docx]

Additional file 5: Comorbidities in patients taking different antihypertensive drugs at 5-year follow-up

| comorbidities | **Total** | **Poor** | **Intermittent** | **Sustained** | ***$\chi^{2}$*** | ***p*-Value** |
| --- | --- | --- | --- | --- | --- | --- |
| All comorbidities, n (%) | 272(21.7) | 76(18.1) | 37(19.3) | 159(24.8) | 7.603 | 0.022 |
| DM, n (%) | 219(17.5) | 51(12.1) | 32(16.7) | 136(21.2) | 14.708 | 0.001 |
| Stroke, n (%) | 19(1.5) | 7(1.7) | 2(1.0) | 10(1.6) | 0.359 | 0.836 |
| CVD, n (%) | 40(3.2) | 19(4.5) | 2(1.0) | 19(3.0) | 5.362 | 0.068 |
| Hyperlipidemia, n (%) | 13(1.0) | 4(1.0) | 2(1.0) | 7(1.1) | 0.050 | 0.975 |
